# The potential effect and mechanism of Saikosaponin A against gastric cancer

**DOI:** 10.1186/s12906-023-04108-3

**Published:** 2023-08-22

**Authors:** Chao Wang, Ruijuan Zhang, Xu Chen, Mengyun Yuan, Jian Wu, Qingmin Sun, Chunrun Miao, Yali Jing

**Affiliations:** 1 China Pharmaceutical University, Nanjing Drum Tower Hospital, Nanjing, 210008 Jiangsu Province China; 2 Clinical Medical College, Nanjing University of Chinese Medicine, Nanjing, 210023 Jiangsu China; 3 Jiangsu Province Hospital of Chinese Medicine, Affiliated Hospital of Nanjing University of Chinese Medicine, Nanjing, 210029 Jiangsu China; 4 Department of Gastroenterology, Dongtai Hospital of Traditional Chinese Medicine, Dongtai, 224299 Jiangsu China; 5 Department of Endocrinology, Drum Tower Hospital Affiliated to Nanjing University Medical School, No. 321 Zhongshan Road, Nanjing, 210008 Jiangsu China

**Keywords:** Network pharmacology, Saikosaponin A, Gastric cancer, Apoptosis

## Abstract

**Background:**

Saikosaponin A (SSA) shows a series of pharmacological activities, such as anti-inflammatory, antioxidant and antitumor. However, there is a lack of comprehensive research or sufficient evidence regarding the efficacy of SSA in treating gastric cancer (GC), and the specific mechanisms by which it inhibits GC growth and progression are still not fully understood.

**Methods:**

MTT and clonogenic assays were employed to detect the effect of SSA on the proliferation of GC cells. Bioinformatics predicted the SSA targets in the treatment of GC. The core genes and the underlying mechanism of SSA in anti-GC were obtained by analyzing the intersecting targets; molecular docking and Western blot were used to check the reliability of core genes. Flow cytometry was used to analyze apoptosis and cell cycle in GC cells treated with varying concentrations of SSA. Western blot was employed to detect the expression levels of related proteins.

**Results:**

SSA significantly blocked GC cells in the S phase of the cell cycle and induced apoptosis to suppress the proliferation of GC cells. Network pharmacology revealed that the underlying mechanisms through which SSA acts against GC involve the modulation of several signaling pathways, including the PI3K-Akt, MAPK, RAS, and T-cell signaling pathways. Molecular docking showed pivotal target genes with a high affinity to SSA, including STAT3, MYC, TNF, STAT5B, Caspase-3 and SRC. Furthermore, western blot results revealed that SSA significantly increased the protein levels of Bax and Cleaved Caspase-3, whereas decreased the expression levels of p-JAK, p-STAT3, MYC, Bcl-2, p-PI3K, p-AKT and p-mTOR, confirming that the reliability of hub targets and SSA could promote GC cell apoptosis by suppressing PI3K/AKT/mTOR pathway.

**Conclusions:**

The results suggest that SSA has the ability to trigger apoptosis in GC cells by blocking the PI3K/AKT/mTOR pathway. These findings highlight the potential of SSA as a promising natural therapeutic agent for the treatment of GC.

**Supplementary Information:**

The online version contains supplementary material available at 10.1186/s12906-023-04108-3.

## Introduction

Gastric cancer (GC) remains the fourth leading reason of cancer related death worldwide according to global cancer statistics in 2020 [1]. Despite significant advances in therapies for gastric cancer, including surgery, chemotherapy, targeted therapy and immune checkpoint inhibitors, the prognosis of gastric cancer is still very poor, causing a large socioeconomic burden, especially in China [2, 3]. Hence, the identification of novel treatment approaches for GC holds great importance.

Natural products have always played a crucial role in the pharmaceutical drugs, particularly in the field of oncology. Most currently available chemotherapy drugs, including paclitaxel, vincristine and vinblastine, are all derived from natural sources [4, 5]. *Radix Bupleuri* (RB) has been used for thousands of years in China, and its injection has also been of clinically useful for more than 80 years, with the disadvantages of complex composition and uncontrollable quality. Saikosaponin A (SSA) is a triterpenoid glycoside extracted from RB. As a major bioactive compound of RB, SSA shows a series of pharmacological activities, such as anti-inflammatory, antioxidant, antiviral and antitumor [6]. However, limited reports have mentioned the effect of SSA against GC cells, and its underlying anti-GC mechanism has yet to be elucidated [6–8]. In recent years, network pharmacology, an emerging area of pharmacology, has revealed multiple targets and mechanisms of drug action by mapping the human disease-gene network onto the polypharmacology network [9, 10]. Network pharmacology provides a new strategy for drug discovery and simultaneously becomes a powerful tool for exploring the mechanisms of natural products [11, 12].

**Fig. 1:**
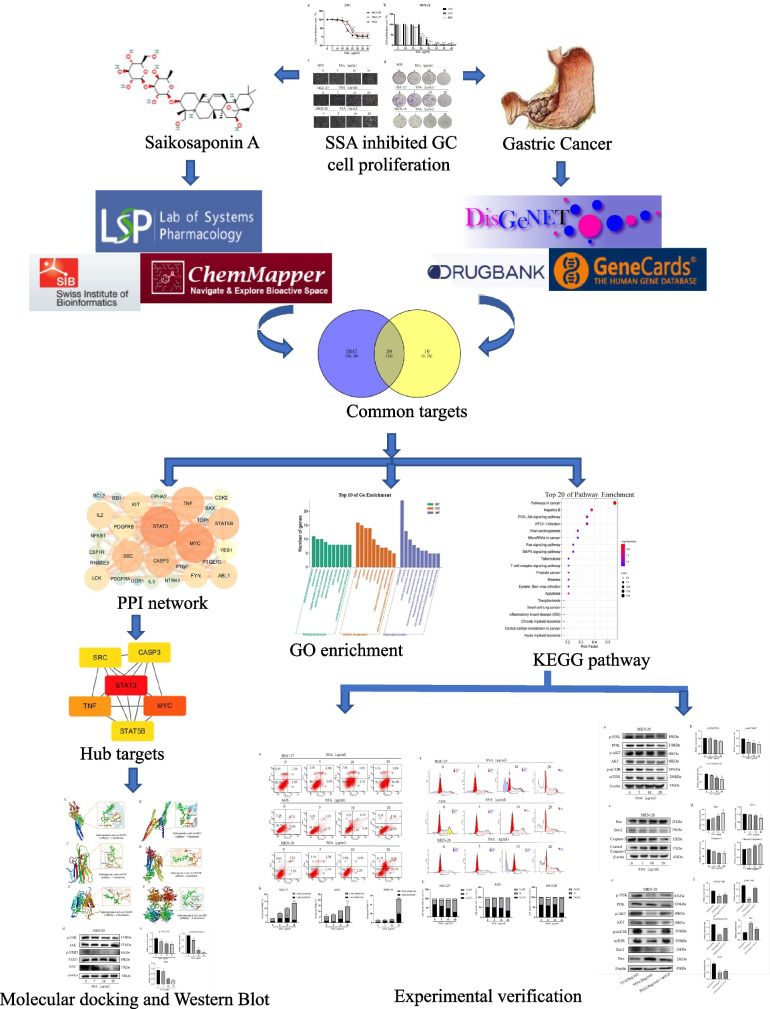
Scheme of the study

In the present study, we first obtained potential common targets to predict the molecular mechanism of SSA against GC using network pharmacology. Subsequently, we showed the effect of SSA on the proliferation, migration, apoptosis and cycle of GC cells (AGS, HGC-27 and MKN-28). We confirmed that SSA induced GC cell apoptosis partly by inhibiting the PI3K/AKT pathway (Fig. 1).

## Materials and methods

### Chemicals and reagents

SSA (purity > 95%) was obtained by Chengdu Must Bio-Technology Co., Ltd. (Chengdu, China) and dissolved in dimethyl sulfoxide (DMSO) at a concentration of 50 mg/ml. Antibodies against p-PI3K (No. 4228S), PI3K (No. 5405S), p-AKT (No. 2965S), AKT (No. 4691S), p-mTOR (No. 2971S), mTOR (No. 2972S), Cleaved Caspase-3 (No. 9554 T) and Bax (No. 2772 T) were acquired from Cell Signaling Technologies (MA, USA). Antibodies against Caspase-3 (No. 66470–2-lg), Bcl-2 (No. 26593–1-AP), p- JAK (No. 4869S), p- STAT (No. 5027S) and MYC (No. 6935S) were purchased from Proteintech Group (MA, USA). Anti-mouse (No. ZB-2305) and anti-rabbit (No. ZB-2301) horseradish peroxidase-conjugated antibodies were acquired from Zhongshanjingqiao Biotechnology (Beijing, China). 740 Y-PTFA (123,618–16-1), a potent and cell-permeable PI3K activator, was purchased from MedChemExpress (MA, USA).

### Cell culture

The human gastric cancer cell lines AGS, HGC-27 and MKN-28 were acquired from the Shanghai Cell Bank of the Chinese Academy of Sciences. All cells were cultured in RPMI 1640 medium (Gibco, USA) supplemented with 10% newborn calf serum (Evergreen Company, Hangzhou, China) and 1% penicillin/streptomycin, and incubated at 37 °C in a humidified atmosphere of 5% CO_2_.

### Cell proliferation assay

The effect of SSA on GC cell proliferation was evaluated by 3-(4,5-dimethylthiazol-2-yl)-2,5-diphenyltetrazolium bromide (MTT; Sigma, MO, USA). The cells were seeded in a 96-well plate at a density of 5000 cells per well. After overnight cultured, the cells were incubated with RPMI 1640 medium containing 0, 5, 10, 15, 20, 25, 30, 35, or 40 μg/ml SSA for 12, 24 and 48 h. Then, the cells were incubated with MTT (120 μl, 5 mg/ml) at 37 °C for 4 h. After removing the supernatant, the cells were incubated with 150 μl of DMSO for 10 min. Cell viability was examined with a microplate reader (Bio-Tek, USA) at a 570 nm absorbance. Ultimately, we selected 0, 5, 10 and 20 μg/ml SSA, and 24 h for further study according to the IC_50_ values at different times.

### Colony formation assay

GC cells (500 cells/well) were seeded in a 6-well plate, treated with RPMI 1640 medium containing 0, 5, 10 and 20 μg/ml SSA for 24 h, and then cultured for 7–10 days. After washing twice with PBS, the cells were stained with 1 ml of paraformaldehyde (Beyotime Biotechnology, Shanghai, China) at 4 °C for 1 h, added to 1 ml of crystal violet (Beyotime Biotechnology, Shanghai, China) for 2 min, washed with water several times, and then photographed.

### Target predictions for SSA and GC

**Fig. 2:**
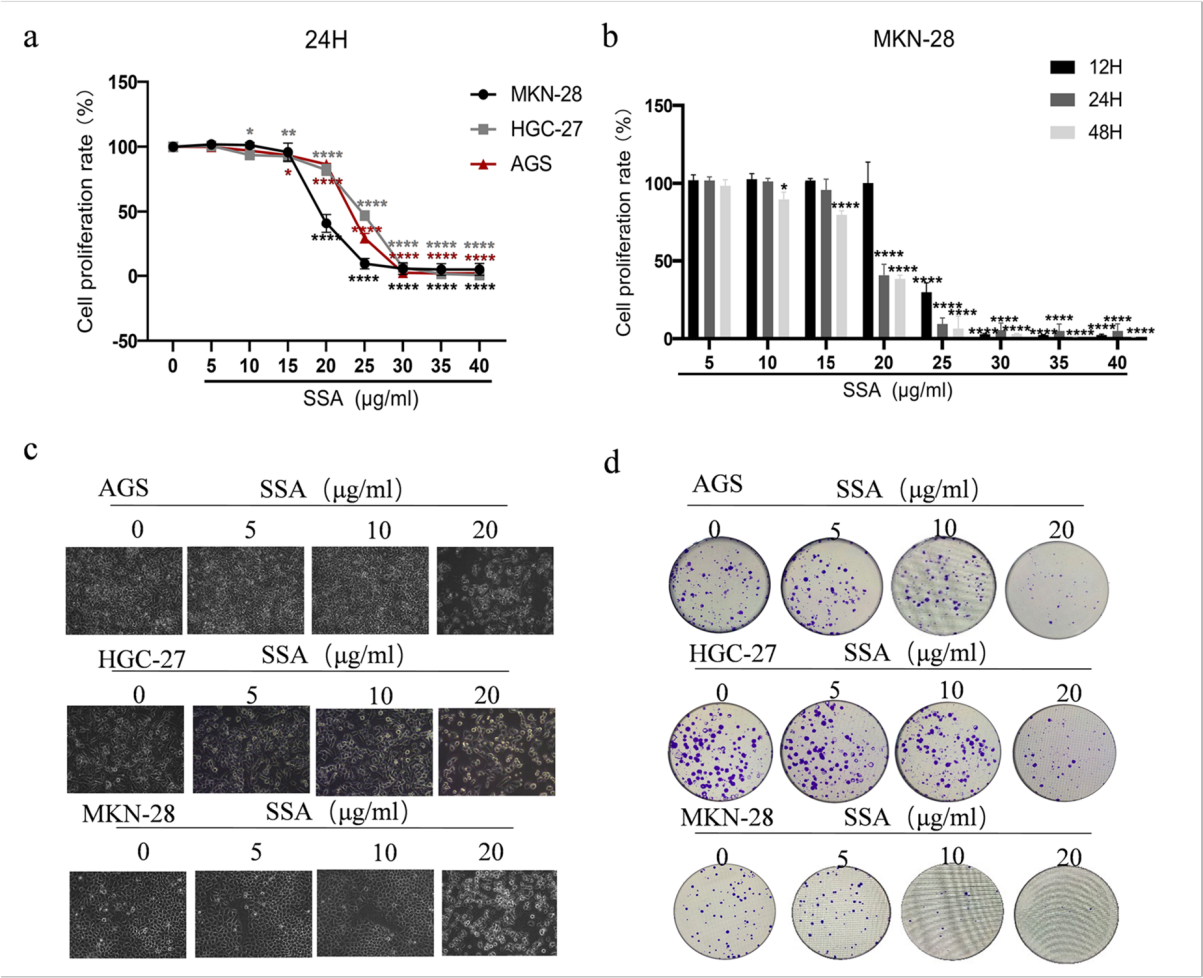
SSA suppressed proliferation in GC cells. **a** AGS, HGC-27 and MKN-28 cells were treated with different concentrations of SSA for 24 h. **b** MKN-28 cells were incubated with SSA for 12, 24 and 48 h. **c** Representative cell morphological changes. Scale bars: 50 μm. **d** Colony formation assay of SSA in AGS, HGC-27 and MKN-28 cells. Data are presented as the mean ± SD (*n* = 3), *P < 0.05, **P < 0.01, ***P < 0.001 compared to the control group

Structural information regarding SSA (Fig. 2a, CAS: 20736–09-8) was acquired from NCBI PubChem (https://pubchem.ncbi.nlm.nih.gov/). The targets of SSA were obtained from the TCMSP database (http://lsp.nwu.edu.cn/tcmsp.php)  [13], Swiss Target Prediction database (http://www.swisstargetprediction.ch/) filtered by the condition of “probability > 0” [14] and Chem mapper database (https://lilab-ecust.cn/chemmapper/) [15] filtered by the condition of “Homo sapiens; score > 0”.

Genes associated with GC were obtained from three public database sources, including the DisGeNET database (https://www.disgenet.org/) [16], DrugBank database (https://go.drugbank.com/) [17], and GeneCards database (https://www.genecards.org/) [18]. Using “Stomach Carcinoma” as the keyword, we searched and screened gastric cancer-related target datasets, and retained “Homo sapiens” proteins linked to GC. Subsequently, we submitted all targets to the UniProt database (http://www.UniProt.org/) [19], and restricted the types to “Homo sapiens” for standardization.

### PPI network establishing and hub target screening

The common targets related to SSA and GC were acquired by Venny 2.1 (http://bioinfogp.cnb.csic.es/tools/venny/index.html) . Afterward, the interaction relationship of common targets was explored by the STRING database (https://string-db.org/) [20] with the condition “Homo sapiens” and score value > 0.4. Subsequently, the PPI network file of common goals was inputted into Cytoscape 3.7.2 for further study. Cytoscape can analyze complex networks with powerful data integration and visualization functions [21]. To discover the highly interconnected regions of interaction in PPI networks, the top 6 proteins based on the degree level were defined as hub targets.

### GO and KEGG pathway enrichment study

Gene ontology (GO) is a commonly used approach for annotating genes and their products from organisms, including analyses of molecular function (MF), biological process (BP), and cellular component (CC) [10]. Kyoto Encyclopedia of Genes and Genomes (KEGG) is a useful database resource that links genomic information with functional information by systematically analyzing gene functions [22–24]. The enrichment analyses of GO terms and KEGG pathways were carried out by the DAVID database (https://david.ncifcrf.gov/), which helps the genes’ functional interpretation [25]. The results of pathway enrichment might show critical mechanism of action for SSA against GC.

### Molecular docking and Western blot verification

The MOL2 format of SSA was downloaded from the PubChem database and then converted to the 3D structure using Open Babel (ver 2.4.1). The crystal structure of the candidate protein was retrieved from the RCSB protein database (http://www.pdb.org/), and the structure was optimized by deleting water molecules, adding hydrogen, and adding charges. Molecular docking calculations were executed by AutoDock Vina 1.1.2, and all docking operation options were default values. Docking scores can predict the binding affinity between molecules and proteins. Finally, the docking results with the highest score were visualized through PyMoL and Western blot.

### Apoptosis analysis

Cell death and apoptosis were assessed using the Annexin V-FITC Apoptosis Detection Kit I (Keygen Biotech, Nanjing, China). MKN-28 cells (5 × 10^5^ cells/well) were inoculated into 6-well plates and cultured overnight. After culturing with 1640 complete medium containing 0, 5, 10 and 20 μg/ml SSA for 24 h, the cells were washed with PBS, centrifugated, resuspended, and collected into a flow cytometry tube. Afterward, the cells were incubated with Annexin V and propidium iodide (PI) for 15 min, and detected by a FACSAria III flow cytometer (BD Biosciences, USA).

### Cell cycle analysis

MKN-28 cells (5 × 10^5^ cells/well) were seeded into 6-well plates, and incubated for 24 h. After treatment with SSA at different concentrations (0, 5, 10, 20 μg/ml) for 24 h, the cells were washed with cold PBS, and then fixed with cold 70% ethanol at 4 °C overnight. After that, a cell cycle analysis kit (Beyotime Biotechnology, Shanghai, China) was used to detect the cell cycle distribution based on the manufacturer’s instruction. Cell cycle distribution was analyzed by a FACSAria III flow cytometer (BD Biosciences, USA) with Modifit software.

### Western blot analysis

SSA (0, 5, 10, 20 μg/ml) were subjected to MKN-28 cells for 24 h, respectively. The cells were lysed with RIPA lysis buffer (containing a protease inhibitor and dephosphorylation inhibitor) on ice, and the supernatant was collected as cell protein extracts. Afterward, the protein concentration was measured with a BCA Protein Assay Kit (Beyotime Biotechnology, Shanghai, China). Protein extracts were separated by Smart PAGE™ Precast Protein Gel (4–20%) (Smart-lifesciences, Changzhou, China) and transferred to PVDF membranes. The membranes were blocked in 5% bovine serum albumin (BSA) for 1 h, and then incubated with the primary antibodies at 4 °C overnight. The molecular weight of the target proteins is approximate, so the partial original blots were cut before hybridizing with the primary antibodies. Then the blots were incubated with an appropriate secondary antibody at room temperature for 1 h. Finally, the protein bands were visualized using an enhanced chemiluminescence (ECL) kit and quantified with a Chemiluminescence Imaging System (ChemiDoc XRS, Bio-Rad). In addition, WB quantifications were performed by the Image Lab system, version 5.1.

### Rescue experiment

SSA (0 μg/ml), SSA (20 μg/ml), 740 Y-PTFA, and 740 Y-PTFA + SSA (20 μg/ml) were cultured with MKN-28 cells for 24 h. Western blot was used to detect the expression of PI3K, p-PI3K, Akt, p-Akt, p-mTOR, mTOR, Bcl-2 and Bax in each group.

### Statistical analysis

Data are represented as the mean ± SD. Comparisons between different groups were used with one-way ANOVA with LSD as a post hoc comparison in GraphPad Prism 8.0 software, and *P* < 0.05 was considered indicative of a statistically significant difference.

## Results

### SSA inhibited GC cell proliferation

We initially evaluated the in vitro effect of SSA in the treatment of GC cell lines HGC-27, AGS and MKN-28. The cells were cultured with SSA at different concentrations for 24 h to calculate the IC_50_ values. The results showed 18.99 μg/ml in MKN-28 cells, 24.73 μg/ml in HGC-27 cells, and 23.41 μg/ml in AGS cells (Fig. 2a), indicating that SSA exerted potent cytotoxicity in GC cells. We also found that SSA decreased GC cell viability in a time and dose-dependent manner (Fig. 2b). Specially, increasing the concentration of SSA led to a gradual decrease in the number of GC cells, accompanied by a change in their morphology towards a rounder shape, suggesting that SSA has the capability to inhibit the growth of GC cells and induce alterations in their normal morphology (Fig. 2c). Furthermore, the colony formation assay also indicated that SSA inhibited the generation of cell colonies in a concentration-dependent manner (Fig. 2d). These data suggested that SSA inhibited GC cell proliferation.

### Screening the hub targets of SSA and GC

**Fig. 3:**
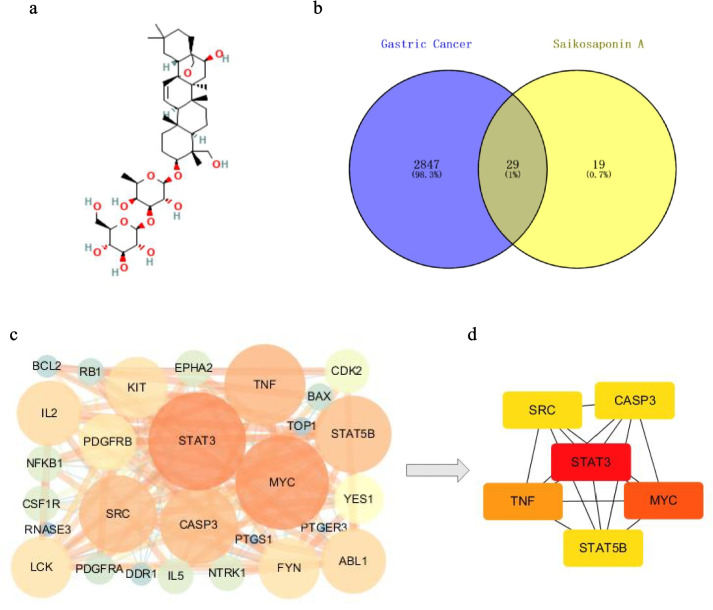
PPI network and hub targets. **a** The chemical structure of SSA. **b** Venn diagram of the intersecting targets for SSA and GC. **c** PPI network of SSA in the treatment of GC. **d** The top 6 hub genes of SSA against GC. The redder and larger the node is, the more important it is in the network

To explore the potential targets of SSA in the proliferation of GC cells, we conducted a screening process to identify the key targets that are common to both SSA and GC. The chemical structure of SSA is presented in Fig. 3a. In the study, we obtained 48 predicted genes associated with SSA and 2876 disease targets related to GC after removing duplicate targets. Subsequently, the targets associated with SSA or GC were imported into Venny 2.1 to acquire 29 common genes (Fig. 3b). Detailed information on the common target genes is shown in Table 1. Next, the PPI network was constructed by the STRING database and visualized with Cytoscape, which composed of 29 nodes and 152 edges (Fig. 3c). The top 6 potential candidate target genes, including signal transducer and activator of transcription 3 (STAT3), Myc proto-oncogene protein (MYC), tumor necrosis factor (TNF), signal transducer and activator of transcription 5B (STAT5B), Caspase-3 (CASP3) and proto-oncogene tyrosine-protein kinase Src (SRC), were identified as hub genes according to the degree value (Fig. 3d). Detailed information about the hub genes is shown in Table 2.

**Table 1:** Potential common targets for SSA in the treatment of GC

No	Uniport ID	Gene	Protein names
1	Q07812	BAX	Apoptosis regulator BAX
2	P10415	BCL2	Apoptosis regulator Bcl-2
3	P51692	STAT5B	Signal transducer and activator of transcription 5B
4	P06239	LCK	Tyrosine-protein kinase Lck
5	P10721	KIT	Mast/stem cell growth factor receptor Kit
6	P00519	ABL1	Tyrosine-protein kinase ABL1
7	P19838	NFKB1	Nuclear factor NF-kappa-B p105 subunit
8	P40763	STAT3	Signal transducer and activator of transcription 3
9	P29317	EPHA2	Ephrin type-A receptor 2
10	P01106	MYC	Myc proto-oncogene protein
11	P24941	CDK2	Cyclin-dependent kinase 2
12	P11387	TOP1	DNA topoisomerase 1
13	Q9UKK6	NXT1	NTF2-related export protein 1
14	P06241	FYN	Tyrosine-protein kinase Fyn
15	P04629	NTRK1	High affinity nerve growth factor receptor
16	Q08345	DDR1	Epithelial discoidin domain-containing receptor 1
17	P42574	CASP3	Caspase-3
18	P01375	TNF	Tumor necrosis factor
19	P07947	YES1	Tyrosine-protein kinase Yes
20	P12931	SRC	Proto-oncogene tyrosine-protein kinase Src
21	P09619	PDGFRB	Platelet-derived growth factor receptor beta
22	P23219	PTGS1	Prostaglandin G/H synthase 1
23	P06400	RB1	Retinoblastoma-associated protein
24	P05113	IL5	Interleukin-5
25	P16234	PDGFRA	Platelet-derived growth factor receptor alpha
26	P12724	RNASE3	Eosinophil cationic protein
27	P43115	PTGER3	Prostaglandin E2 receptor EP3 subtype
28	P60568	IL2	Interleukin-2
29	P07333	CSF1R	Macrophage colony-stimulating factor 1 receptor

**Table 2:** Detailed information of hub genes

Name	Degree	Betweenness centrality	Closeness centrality
STAT3	23	0.11907383	0.87096774
MYC	22	0.10114926	0.84375
TNF	19	0.20020165	0.77142857
STAT5B	18	0.03213145	0.75
CASP3	18	0.054847	0.75
SRC	18	0.04591592	0.75

### Prediction of the mechanism of SSA against GC

**Fig. 4:**
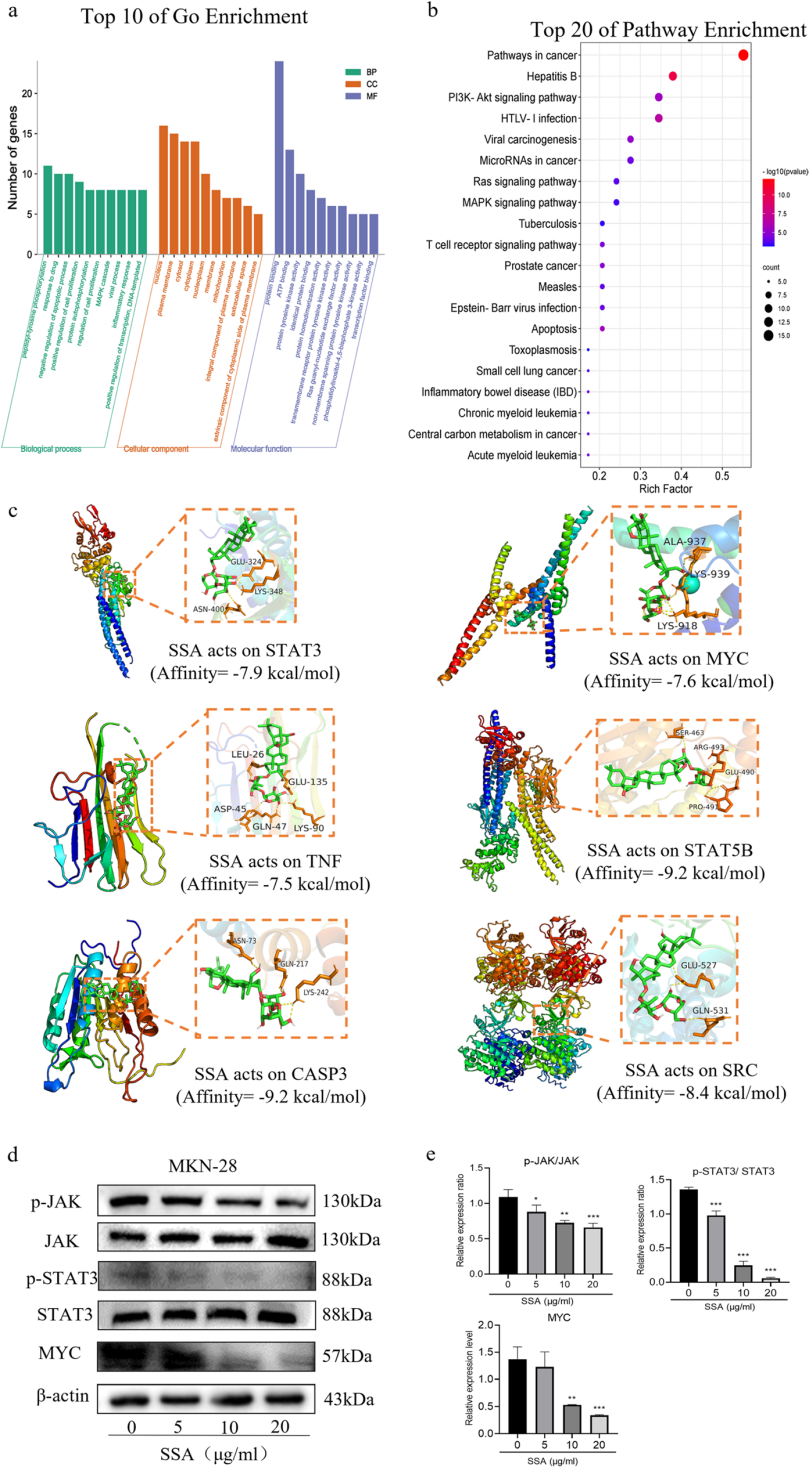
Enrichment analysis of common target genes and verification of hub targets. **a** GO functional enrichment analysis involving BP, CC and MF categories. **b** KEGG pathway enrichment analysis. The size and color of the dots represented the number of genes and the corrected P value, respectively. c Docking results of SSA with the hub targets STAT3, MYC, TNF, STAT5B, CASP3, and SRC. **d** The protein expression levels of p-JAK, JAK, p- STAT3, STAT3 and MYC in MKN-28 cells after SSA treatment. **e** The relative protein expression of p-JAK/JAK, p-STAT3/STAT3 and MYC in MKN-28 cells after SSA treatment

To further probe the underlying mechanism of SSA in the treatment of GC, GO functional and KEGG pathway enrichment analyses of 29 common targets were carried out by the DAVID database. According to GO enrichment analysis, BP was significantly enriched in “response to drug”, “negative regulation of cell proliferation process” and “regulation of apoptotic”. CC was enriched in “nucleus”, “plasma membrane” and “cytosol”. The MF was mainly associated with “transmembrane receptor protein tyrosine kinase activity”, “protein tyrosine kinase activity” and “ATP binding” (Fig. 4a). GO enrichment analysis indicated that SSA could participate in various cellular anticancer processes.

Meanwhile, the enrichment analysis of KEGG pathway showed a total of 55 signaling pathways with significance that were strongly related to “pathways in cancer”, “PI3K-Akt signaling pathway”, “Ras signaling pathway”, “MAPK signaling pathway”, “T-cell receptor pathway”, “apoptosis”, etc. (Fig. 4b). Among them, the PI3K-Akt signaling pathway corresponded to the most targets, including 10 potential therapeutic targets. The PI3K-Akt pathway is a critical signaling pathway for cell survival that regulates cell proliferation and motility, and activation of this pathway can lead to abnormal cell proliferation in GC [26]. In addition, triggering apoptosis is a common strategy in anticancer therapies [27]. Therefore, we speculated that the mechanism of SSA in the treatment of GC can partly induce cell apoptosis by regulating the PI3K-Akt signaling pathway. Moreover, to validate the reliability of the protein–ligand interactions, molecular docking was carried out for 6 hub proteins and SSA. In molecular docking, the affinity score is a measure of the binding strength between molecules. A lower score indicates a stronger binding affinity. A score < -5.0 kcal/mol suggested good binding activity between the ligand and targets [28]. The docking scores of the hub target proteins and SSA are shown in Table 3. The results indicated that the 6 hub targets had a better binding activity to SSA (Fig. 4c).

**Table 3:** Vina scores and cavity information of the hub targeted proteins

No	Receptor	PDB ID	Binding sites with the amino acid	Autodock score (kcal/mol)
1	STAT3	6QHD	GLU-324, LYS-348, ASN-400	-7.9
2	MYC	1EE4	ALA-937, LYS-939, LYS-918	-7.6
3	TNF	5MU8	LEU-26, GLU-135, ASP-45, GLN-47, LSY-90	-7.5
4	STAT5B	6MBW	SER-463, ARG-493, GLU-490, PRO-491	-9.2
5	CASP3	6X8I	ASN-73, GLN-217, LYS-242	-9.2
6	SRC	6E6E	GLU-527, GLN-531	-8.4

To validate the aforementioned findings, we measured the expression levels of JAK/STAT/MYC proteins. The results showed that SSA decreased p-JAK, p-STAT3 and MYC levels in MKN-28 cells (Fig. 4d-e). These findings suggest that the inhibition of MKN-28 cell proliferation by SSA is achieved through the regulation of JAK/STAT/MYC levels. Moreover, these results provide further evidence for the reliability of the identified hub targets.

### SSA induced apoptosis and cell cycle S phase arrest in GC cells

**Fig. 5:**
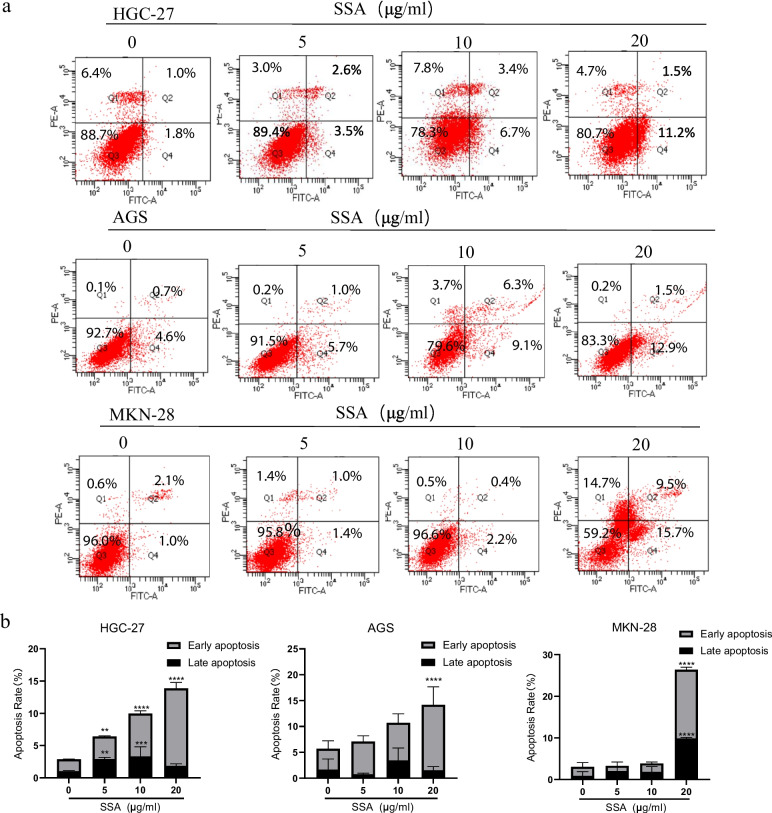
SSA promoted apoptotic death in GC cells. **a** The apoptotic status of HGC-27, AGS and MKN-28 cells at different concentrations of SSA. **b** The percentages of apoptotic cells. Data are presented as the mean ± SD (*n* = 3), *P < 0.05, **P < 0.01, ***P < 0.001 compared to the control group

We further determined whether SSA inhibited GC cell growth via apoptosis. Annexin-V/PI double staining showed that early apoptotic rates were significantly increased in GC cells, especially in MKN-28 cells, following treatment with SSA (Fig. 5). These data suggested that SSA could induce early apoptosis in GC cells in a dose-dependent manner, and the primary pathway of GC cell death induced by SSA is apoptosis. At the same time, we found that the proportion of apoptosis in MKN-28 cells at 20 μg/ml was still about 25%, and the necrosis ratio was about 15%, indicating other ways of SSA inducing GC cell death, which is worth further exploration in more experiments.

**Fig. 6:**
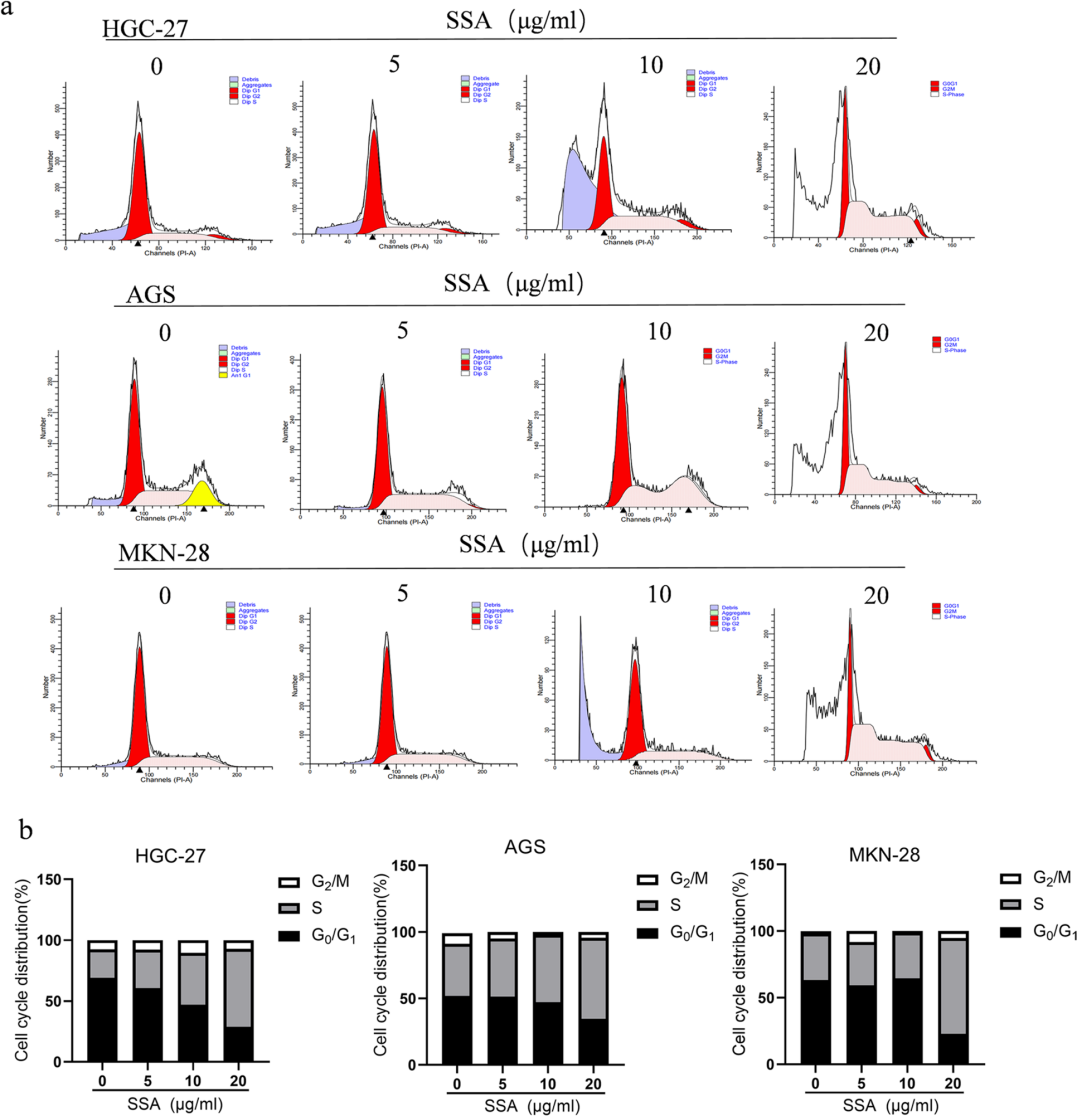
SSA arrested the cell cycle at the S phase in GC cells. a The cell cycle status of HGC-27, AGS and MKN-28 cells treated with different concentrations of SSA. b The percentages of cell cycle cells

To explore the influence of SSA on cell cycle progression in GC cells, a cell cycle assay was performed. After 24 h of SSA treatment, the proportion of GC cells in S-phase significantly increased, while that in G_0_/G_1_ phase decreased (Fig. 6). The results indicated that SSA suppressed GC cell proliferation by blocking the cell cycle in the S-phase.

### SSA induced GC cells apoptosis by the PI3K-AKT pathway

**Fig. 7:**
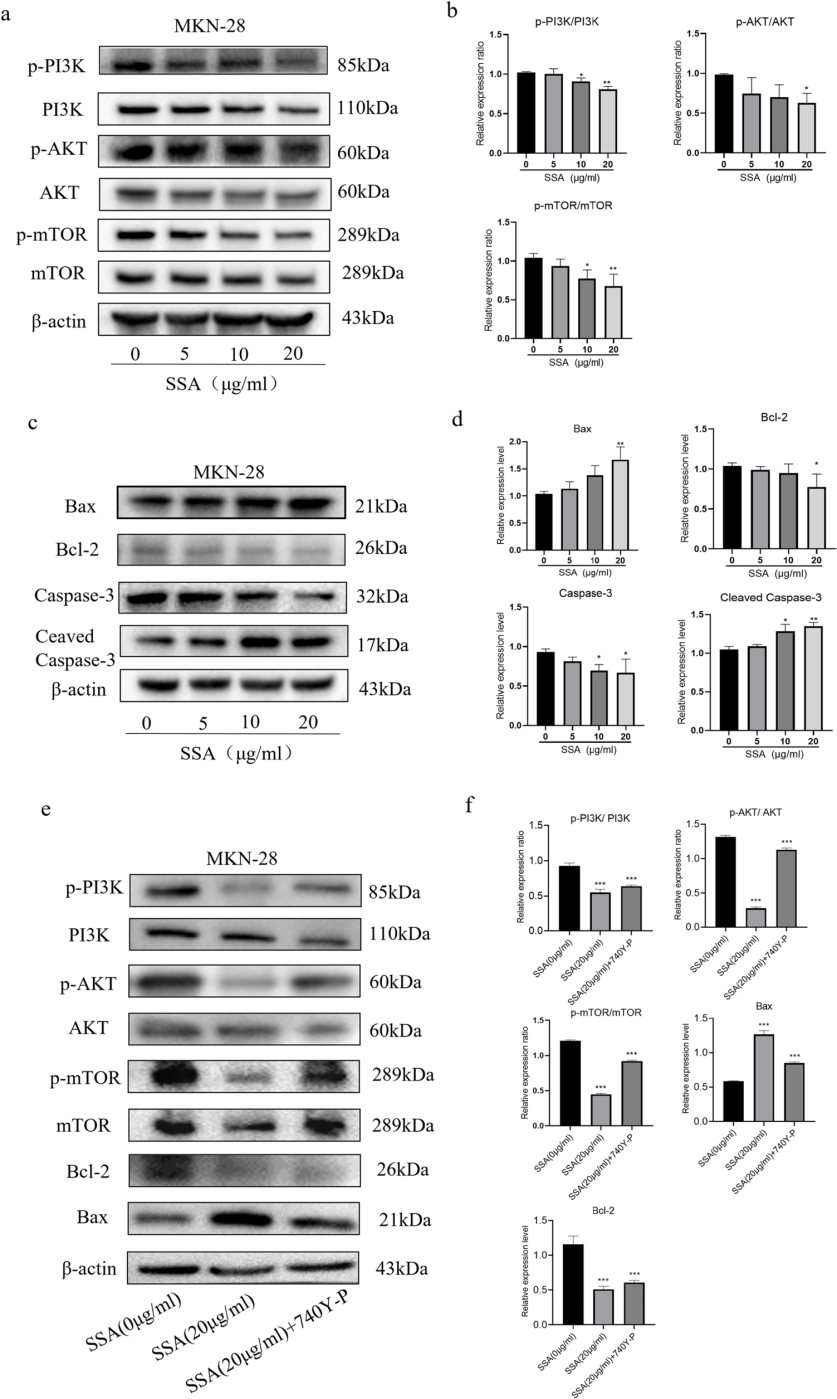
SSA inhibited apoptosis of MKN-28 cells by regulating the PI3K/AKT/mTOR pathway. **a** The protein expression levels of PI3K, p-PI3K, AKT, p-AKT, mTOR and p-mTOR in MKN-28 cells after SSA treatment. **b** The relative protein expression of p-PI3K/PI3K, p-AKT/AKT and p-mTOR/mTOR in MKN-28 cells after SSA treatment. **c** The protein expression levels of Bax, Bcl-2, Caspase-3, and Cleaved Caspase-3 in MKN-28 cells after SSA treatment. **d** The relative protein expression of Cleaved Caspase-3, Caspase-3, Bax and Bcl-2 was normalized to β-actin in MKN-28 cells after SSA treatment. **e** The protein expression levels of PI3K, p-PI3K, AKT, p-AKT, mTOR, p-mTOR, Bax and Bcl-2 in the rescue experiment. **f** The relative protein expression of p-PI3K/PI3K, p-AKT/AKT, p-mTOR/mTOR, Bax and Bcl-2 in the rescue experiment. Data are presented as the mean ± SD (*n* = 3), *P < 0.05, **P < 0.01, ***P < 0.001 compared to the control group. The Western blots have been cropped

Network pharmacology indicated that SSA induced the apoptosis of GC cells by partly regulating the PI3K-AKT pathway. Aberrant activation of the PI3K/Akt/mTOR signaling pathway promotes GC progression through inhibition of apoptosis, drug resistance, angiogenesis, metastasis and epithelial to mesenchymal transition. And clinical trials indicated that inhibition of this pathway could lead to regression of GC [26, 29]. Subsequently, we specifically chose MKN-28 cells that exhibited the most notable apoptotic effect to conduct additional experiments and further validate the hypothesis. Western blot analysis suggested that SSA reduced the protein expression levels of p-PI3K/PI3K, p-AKT/AKT and p-mTOR/mTOR in MKN-28 cells (*P* < 0.05) (Fig. 7a-b). Furthermore, the expression levels of apoptosis-related proteins were also measured. The results showed that SSA markedly enhanced the protein expression of Bax, while reducing the expression levels of Bcl-2 and Cleaved Caspase-3 (*P* < 0.05) (Fig. 7c-d). Additionally, the trend of the difference was more noticeable with the increasing concentration of SSA. These results indicated that SSA induced GC cell apoptosis by partly inhibiting the PI3K/AKT pathway. The rescue experiment also supported this point (Fig. 7e-f).

## Discussion

In this study, we investigated the core targets and potential mechanism of SSA in the treatment of GC via network pharmacology, molecular docking and in vitro experiments. We found that SSA induced GC cell apoptosis by partly inhibiting the PI3K/AKT pathway.

According to the PPI network, STAT3, MYC, TNF, STAT5B, Caspase-3, and SRC were identified as hub nodes. STAT3 is a cytoplasmic transcription factor highly expressed in gastric cancer tissues [30], and its aberrant activation can promote tumor formation by regulating tumor cell proliferation, angiogenesis, migration and immune evasion [31]. MYC plays a valuable role in almost every stage of oncogenesis by orchestrating proliferation, differentiation and metabolism, and its blockade has been widely explored for the treatment of cancer [32]. According to research, TNF has higher levels in patients with GC [33]. STAT5B is an oncology target with a high value for pharmacologic intervention [34]. Caspase-3 is the terminally executed protease in apoptosis, whose activation commits the cell to enter the irreversible fate of apoptosis [35]. SRC is a nonreceptor tyrosine kinase that can promote oncogenesis advancement by regulating cellular proliferation and angiogenesis [36]. It was reported that the expression and activity of SRC are increased in GC. Furthermore, selective SRC inhibitors are entering the clinic to enrich the family of anticancer agents [37]. These hub targets were closely associated with the occurrence or treatment of GC and showed a better affinity for SSA in molecular docking, suggesting the potential effect against GC.

Among them, STAT3 and MYC were the most two common targets, which are typically in the JAK-STAT pathway. Therefore, we detected the levels of JAK/STAT/MYC, and found that SSA can inhibit MKN-28 cell proliferation by regulating JAK/STAT/MYC levels, indicating the reliability of core targets.

KEGG analysis suggested that PI3K-Akt, RAS, MAPK signaling, T-cell receptor, and apoptosis pathways may be involved in the mechanisms by which SSA against GC. It is well known that PI3K-Akt signaling can regulate cell proliferation to promote tumorigenesis [38, 39]. Apoptosis is an ordered process of cell death occurring under physiological or pathological conditions [40]. Dysregulation of apoptosis is one of the main causes of human cancer. Most anticancer therapies aim to trigger apoptosis to eliminate malignant cells [27]. In our study, we revealed that SSA could induce apoptosis of GC cells partly by regulating the PI3K-AKT signaling pathway.

Our results also indicated that SSA could block the cell cycle in the S-phase and induce cell apoptosis to inhibit the proliferation of GC cells. By Western blot experiments, we verified the changes of PI3K-Akt signaling pathway and the levels of the apoptosis-related proteins Bcl-2, Bax and Cleaved Caspase-3 after SSA treatment. Bcl-2 family members and Caspases are key effectors of apoptosis. In the Bcl family of proteins, Bcl-2 exerts an anti-apoptotic effect by blocking the release of cytochrome-c, while Bax shows a pro-apoptotic role by promoting the release of cytochrome-c. The balance between Bcl-2 and Bax regulates the apoptosis state of cells [41]. Caspase-3 is a downstream enzyme that executes the process of cell apoptosis, and is also the most important regulator of apoptosis. When Caspase-3 zymogen is cleaved by protease, Caspase-3 is activated (Cleaved Caspase-3) and expands the protease cascade, eventually leading to nuclear apoptosis [42]. Our results showed that SSA significantly reduced the levels of p-PI3K, p-AKT, p-mTOR, Bcl-2 and Cleaved Caspase-3, and markedly increased the levels of Bax in GC cells. These results suggested that SSA inhibited the PI3K-AKT pathway and regulated Cleaved Caspase-3, Bax and Bcl-2 proteins to promote GC cell apoptosis.

PI3K-AKT signaling pathway, one of the most common dysregulated molecular pathways in human cancer, could block cell death to induce the occurrence of cancer by activating different downstream effectors [43]. It has been reported that many PI3K/Akt/mTOR small molecule inhibitors are widely used in preclinical studies, and some inhibitors, such as idelalisib, duvelisib and deforolimus, have been clinically approved for cancer treatment [26]. In the present study, we also showed that SSA could induce GC cell apoptosis by inhibiting the PI3K/Akt/mTOR signaling pathway, suggesting the potential clinical application of SSA for GC.

## Conclusions

In summary, our results indicated that SSA could induce apoptosis of GC cells partly by suppressing the PI3K/Akt/mTOR signaling pathway. SSA, as a natural compound, has the potential to be developed as a novel multitarget drug for GC treatment. Further experiments are still needed to explore the potential mechanism of SSA in the field of GC therapy.

### Supplementary Information

**Additional file 1:** **Supplementary Table 1.** The information of SSA-related targets. **Supplementary Table 2.** The information of GC-related targets. **Supplementary Table 3.** The KEGG terms of therapy target genes and their corresponding count, corrected p-values, and gene count.

**Additional file 2:** **Supplementary Figure 1.** The effect of SSA on migration of GC cells. 

**Additional file 3.** Original WB Information.

## Data Availability

The datasets used or analyzed during the current study are available from the corresponding author on reasonable request.

## Abbreviations

GC: Gastric cancer
SSA: Saikosaponin A
RB: Radix Bupleuri
MTT: 3-(4,5-Dimethylthiazol-2-yl)-2,5-diphenyltetrazolium bromide
GO: Gene ontology
KEGG: Kyoto Encyclopedia of Genes and Genomes
MF: Molecular function
BP: Biological process
CC: Cellular component
PI: Propidium iodide
ECL: Enhanced chemiluminescence
STAT3: Signal transducer and activator of transcription 3
MYC: Myc proto-oncogene protein
TNF: Tumor necrosis factor
STAT5B: Signal transducer and activator of transcription 5B
CASP3: Caspase-3
SRC: Proto-oncogene tyrosine-protein kinase Src
