# Supplementary material for: The potential effect and mechanism of Saikosaponin A against gastric cancer
Source: BMC Complement Med Ther. 2023 Aug 22;23:295. doi: 10.1186/s12906-023-04108-3 (PMC10463516; doi:10.1186/s12906-023-04108-3)
Supplement: Supplementary file 2 — Additional file 2: Supplementary Figure 1. The effect of SSA on migration of GC cells. [file 12906_2023_4108_MOESM2_ESM.pdf]

Supplementary Material 2

Supplementary Figure 1: The effect of SSA on migration of GC cells

There was no significant difference in migration rate when GC cells were incubated with SSA at different concentrations for 24 hours compared with normal controls ($p > 0.05$). This suggested that SSA does not affect the migration of GC cell lines.

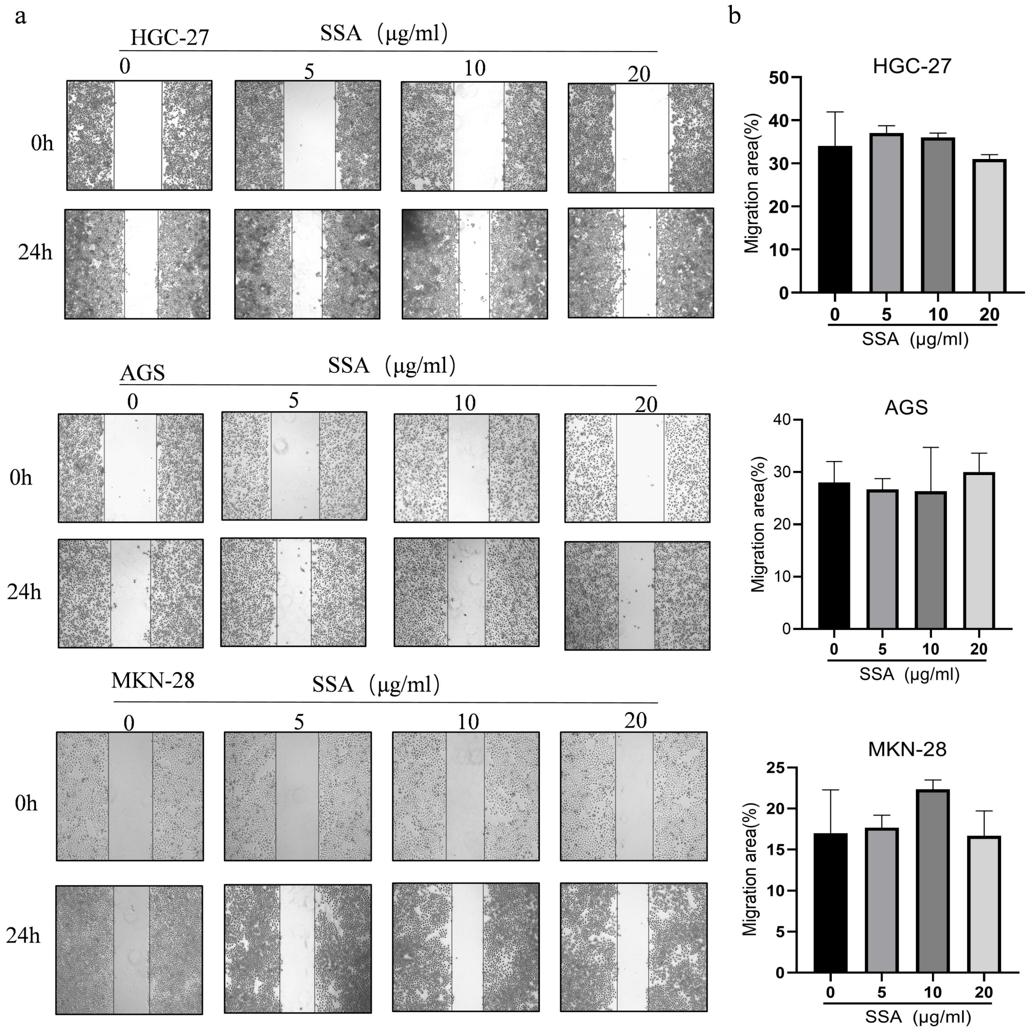

Supplementary Figure 1: The effect of SSA on migration of GC cells. (a) Representative results of wound healing assay of SSA on MKN-28, HGC-27, AGS cells. Scale bars:100 μm. (b) Migration area of wound healing assay of SSA on MKN-28, HGC-27, AGS cells. Data were presented as means ± SD (n=3).
