# Supplementary material for: The potential effect and mechanism of Saikosaponin A against gastric cancer
Source: BMC Complement Med Ther. 2023 Aug 22;23:295. doi: 10.1186/s12906-023-04108-3 (PMC10463516; doi:10.1186/s12906-023-04108-3)
Supplement: Supplementary file 3 — Additional file 3. Original WB Information. [file 12906_2023_4108_MOESM3_ESM.docx]

Original WB Information

FIG4 (d) The protein expression levels of p-JAK, JAK, p- STAT3, STAT3 and MYC in MKN-28 cells after SSA treatment.

**SSA（μg/ml）**

**0**

**5**

**10**

**20**


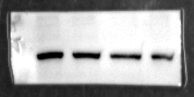

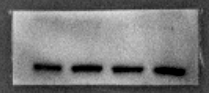


**p-JAK———**

**130KDa**

**JAK———**

**130KDa**

**SSA（μg/ml）**

**0**

**5**

**10**

**20**


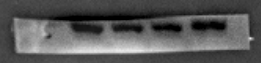

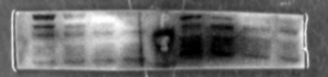


**p-STAT3———**

**88KDa**

**STAT3———**

**88KDa**

**SSA（μg/ml）**

**0**

**5**

**10**

**20**


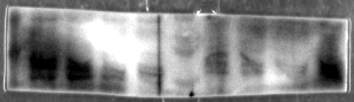

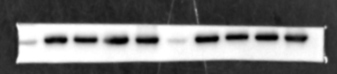


**57KDa**

**MYC———**

**42KDa**

**β-actin———**

FIG.7(a) Original WB of PI3K, p-PI3K, AKT, p-AKT, mTOR, and p-mTOR in MKN-28 cells after SSA treatment.

**SSA（μg/ml）**

**0**

**5**

**10**

**20**


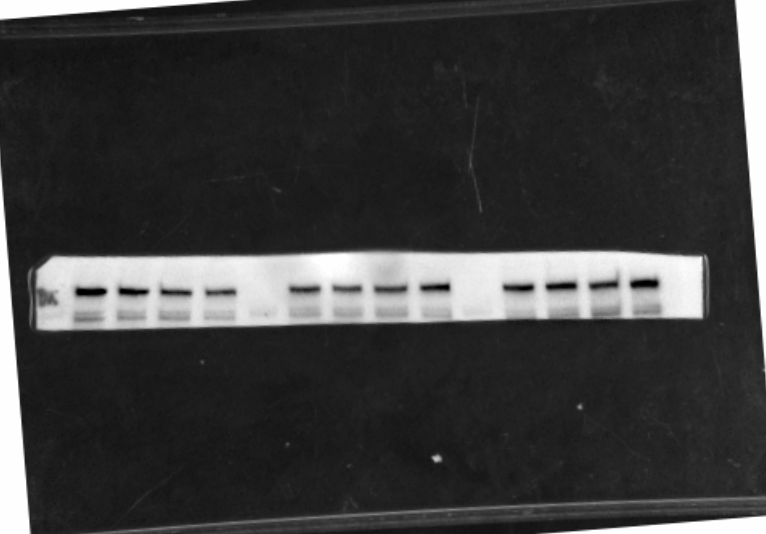

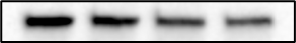


**———180kD**

**p-mTOR 289kD———**

**SSA（μg/ml）**

**0**

**5**

**10**

**20**


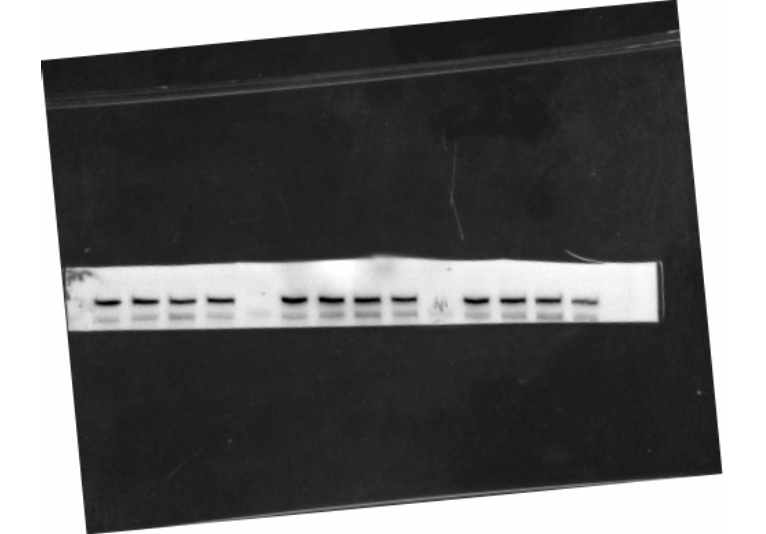


**———180kD**

**mTOR 289kD—————**

**SSA（μg/ml）**

**0**

**5**

**10**

**20**


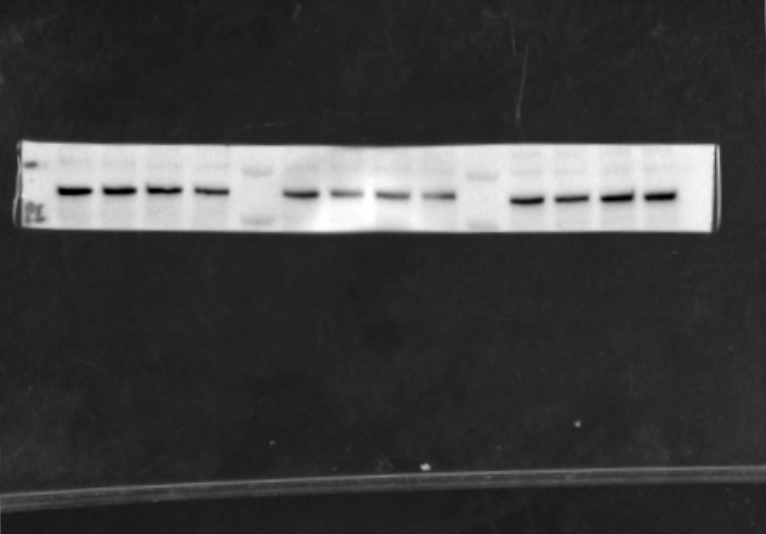


**——130kD**

**Pi3K 110kD————**

**——100kD**

**SSA（μg/ml）**

**0**

**5**

**10**

**20**


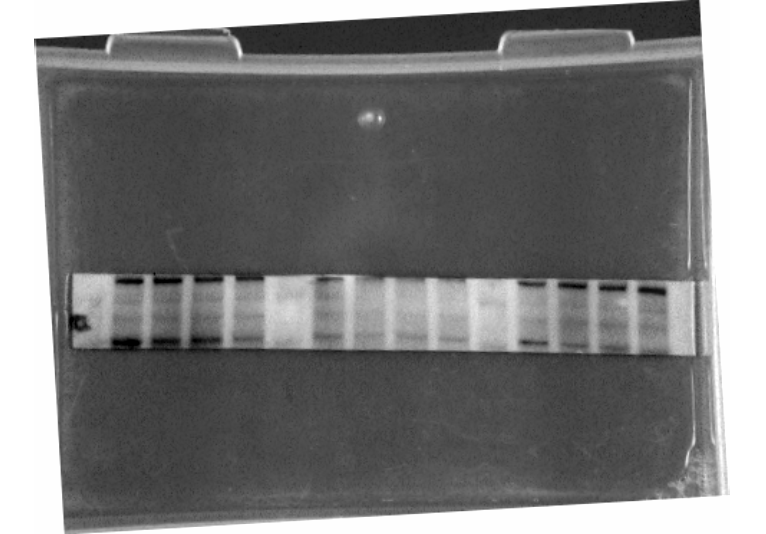


**——100kD**

**——70kD**

**p-Pi3K 85kD————**

**SSA（μg/ml）**

**0**

**5**

**10**

**20**


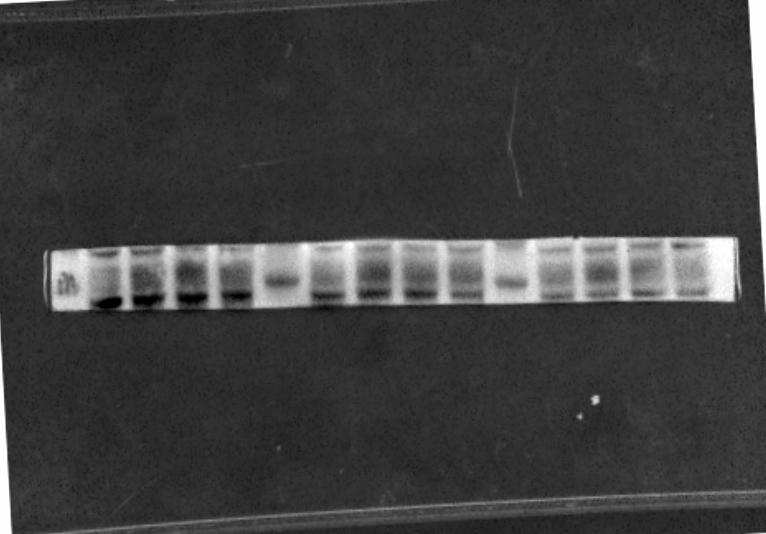


**——70kD**

**p-AKT 60kD————**

**SSA（μg/ml）**

**0**

**5**

**10**

**20**


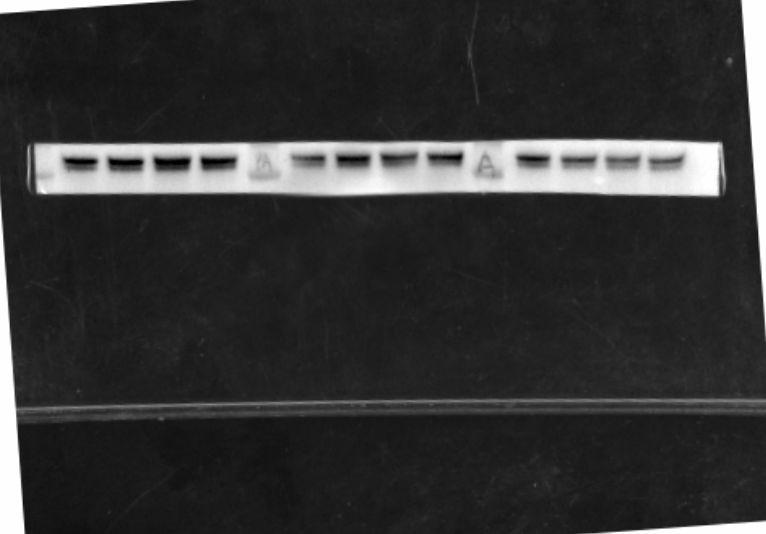


**Akt 60kD————**

**——55kD**

55kDa

**SSA（μg/ml）**

**0**

**5**

**10**

**20**


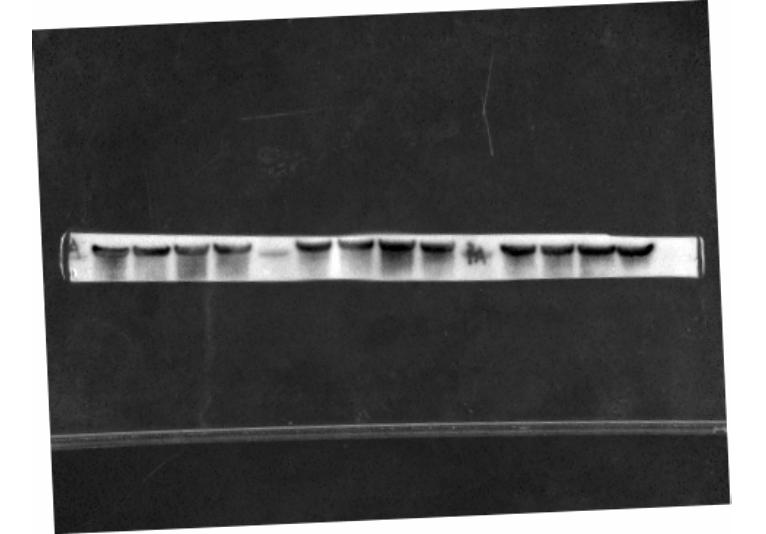


**40kD——**

**β-action 43kD————**

FIG.7(c) Original WB of Bax, Bcl-2, caspase-3, and cleaved-caspase 3 in MKN-28 cells after SSA treatment.

**SSA（μg/ml）**

**0**

**5**

**10**

**20**


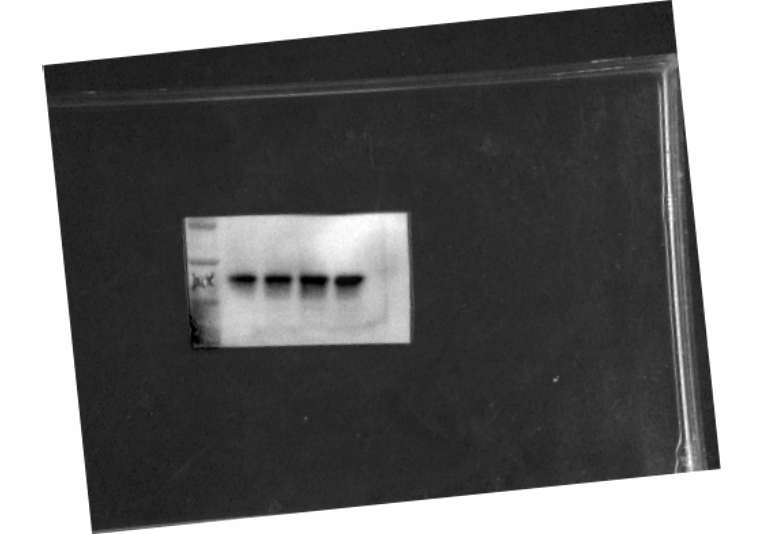


**——25kD**

**Bax 21kD————**

**——15kD**

**SSA（μg/ml）**

**0**

**5**

**10**

**20**


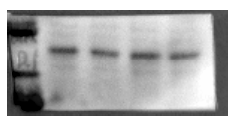


**——35kD**

**Bcl-2 26kD————**

**——25kD**

**SSA（μg/ml）**

**0**

**5**

**10**

**20**


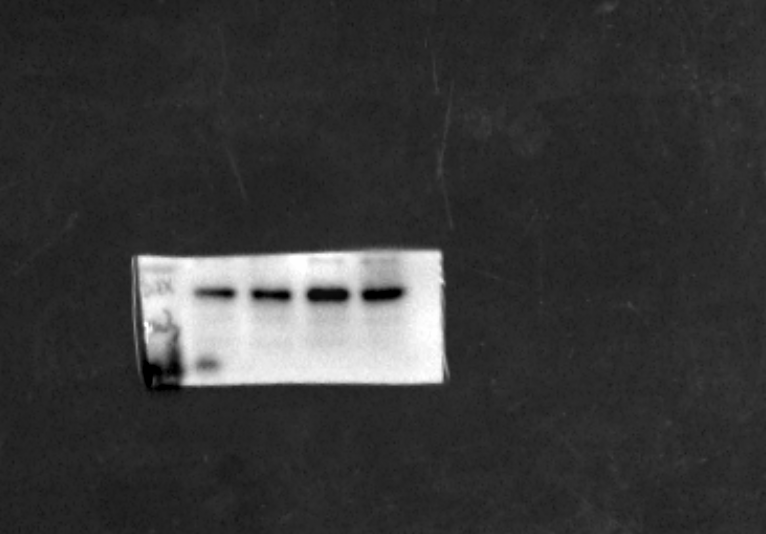


**——25kD**

**Ceaved Caspase-3 17kD————**

**——10kD**

**SSA（μg/ml）**

**0**

**5**

**10**

**20**


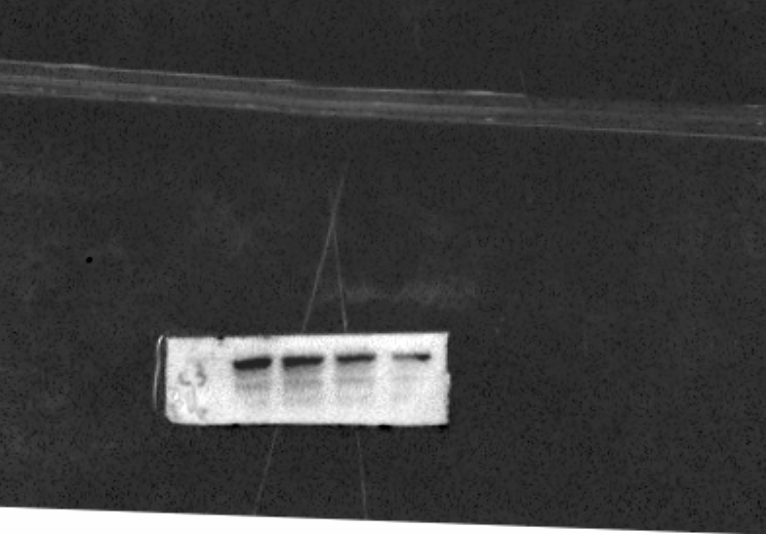


**Caspase-3 32kD————**

**SSA（μg/ml）**

**0**

**5**

**10**

**20**


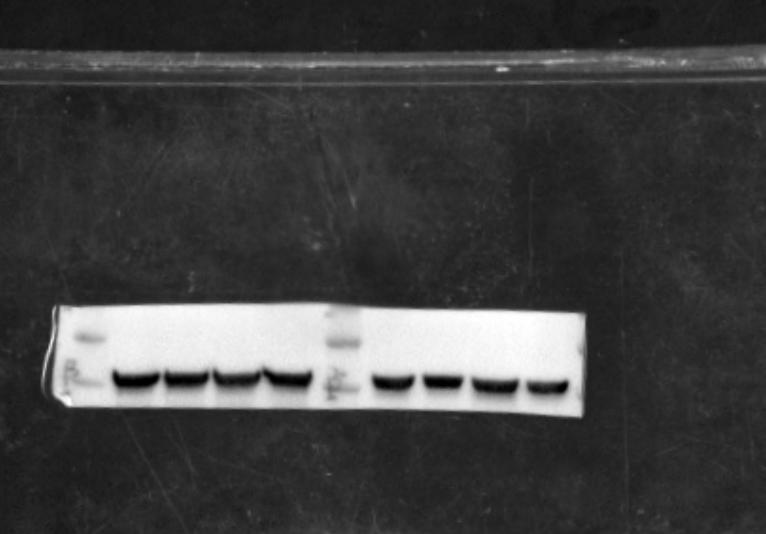


**——55kD**

**——40kD**

**β-action 43kD————**

FIG.7(e) Original WB of PI3K, p-PI3K, AKT, p-AKT, mTOR, p-mTOR, Bax and Bcl-2 in the rescue experiment.

**SSA(20μg/ml)+740Y-P**


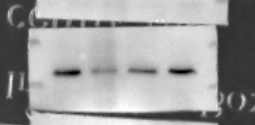

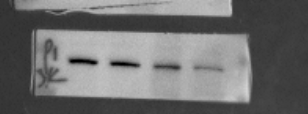


**SSA(20μg/ml)**

**SSA(0μg/ml)**

**85KDa**

**p-PI3K———**

**110KDa**

**PI3K———**


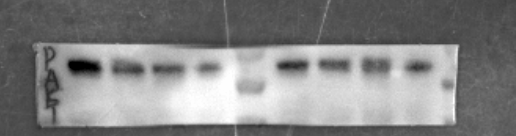

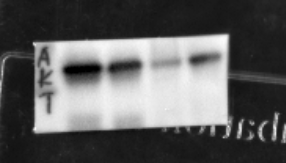


**SSA(20μg/ml)+740Y-P**

**SSA (20μg/ml)**

**SSA(0μg/ml)**

**60KDa**

**p-AKT———**

**60KDa**

**AKT———**


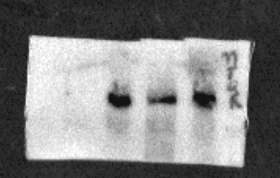

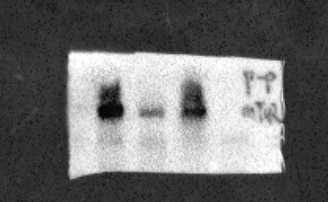


**SSA(20μg/ml)+740Y-P**

**SSA(20μg/ml)**

**SSA(0μg/ml)**

**289KDa**

**p-mTOR———**

**289KDa**

**mTOR———**


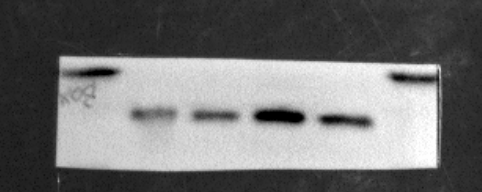

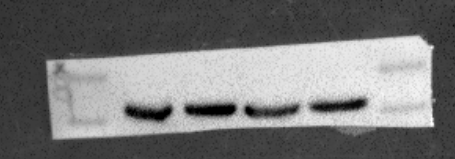


**β**-**actin———**

**Bax———**

**SSA(0μg/ml)**

**SSA(20μg/ml)**

**SSA(20μg/ml)+740Y-P**

**21KDa**

**42KDa**
